# Teaching the Deaf and Dumb to Speak

**Published:** 1870-10

**Authors:** David Greenberger


ARTICLE VI. — *Teaching the Deaf and Dumb to Speak.* Communicated by DAVID GREENBERGER, Ph. D.

At the regular meeting of the Board of Education held on Tuesday the 6th inst., the Superintendent reported that room No. 3, in the La Salle Street school, was given to Mr. Greenberger for the purpose of educating deaf and dumb children between the ages of six and twelve years, after the European system, that is, to teach them to speak and to understand what is spoken to them by distinguishing words from the motions of the speaker's lips.

Almost all parents of deaf mutes in this city and the surrounding places avail themselves of the benefits of this school, which was opened on Monday the 12th inst.

This system of educating deaf mutes—called the Method of Articulation—which has been in use for a long series of years in all schools for that unfortunate class in nearly all Europe, and was recently adopted in the most prominent institutions in this country, is founded upon the fact that speech, or articulation, is a mere mechanical process, the result of certain movements, identical in all individuals, of the lips, tongue, and other vocal organs. A single glance into the looking-glass is sufficient for anybody to perceive that the shape which the mouth assumes in enunciating the vowel *o* is quite different from that which it shows when we pronounce the letters *b*, *d*, etc.

Through patient study and practice a deaf mute acquires the ability of comprehending these visible movements of the organs of speech—technically called lip-reading—with such precision and correctness that we can speak to him just as to persons endowed with the sense of hearing. Many a one who became deaf in old age, can converse by watching closely the lips of the speaker.

That is one part of the system; the other is to enable the deaf mute to utter sounds himself, and this rests upon the fact that the above-named movements of our vocal organs, while performing their office, are necessarily and indispensably accompanied by a more or less forcible emission of breath from the mouth, or a vibration of the throat and chest, caused by the straining of certain muscles located in these parts of the body. While pronouncing the letter *p*, you must feel the puff of air on the back of your hand, if you hold it close before your mouth; or placing your hand under the angle of the jaw, and uttering an *e*, the vibration there is most sensibly felt. Of this vibration, and the expulsion of air from the lips, the sense of feeling renders the deaf mute cognizant, and gives him an idea of sound and speech. Having once that idea conveyed to him, there is no reason why he should not be able to imitate sounds and speech, and learn his native tongue. He does not use it now, not on account of his vocal machinery being incapable of operation, but simply for the same reason that an American child does not use the Chinese or any other foreign language, that is, because he never heard anything about it. He is dumb, because he is deaf. His vocal organs being in quite a normal condition, it requires only to convey an idea of sound to him, and to give him knowledge of his faculty to imitate it.

In the limited space now at our disposal, it is impossible to explain clearly and precisely the mechanical formation of all the letters of the alphabet; considering however that this journal is intended for a highly educated and intelligent class, we think it will be sufficient to have given a few striking examples to enable the reader to comprehend how the deaf mute is taught to utter the sounds of the different letters. As soon as he has learned those, we require him to join them to syllables and then to words with easy pronunciation; first, monosyllables: as, *eye*, *pie*, *tea*, *key*, etc.; then words with two syllables: as, *mamma*, *papa*, *baby*, *lady*, and so on.

Those who are unacquainted with the arduous and intricate labors that devolve upon the deaf mute teacher, generally think that after these mere mechanical exercises are over, the greatest obstacles are surmounted and all the rest of the instruction is a mere trifle. If it were so, the lot of those teachers would be more enviable than it really is. It is now that the difficulties of their task begin—difficulties no layman ever dreams of; for after the idea of the phonetic part of speech has been instilled into the mind of the deaf mute, the most philosophical task begins to make him understand the logical meaning of all the parts of speech, the construction of sentences, the nice shades of meaning expressed in synonymes, etc., etc. Hearing people do not know themselves how they come by all these things, their teachers never had the trouble of explaining to them the meaning of such words as *is*, *has*, *can*, etc. If you do not ask them, they know what those words signify, but if you do ask them, then they do not know. The deaf mute being deprived of one of the most important avenues that convey ideas to the mind, he forms in the same images of only such objects as can be seen with the eyes, or grasped with the hands, etc., everything else is *terra incognita* to him; when he enters school, therefore, he can very easily be taught the names of objects by pointing at them or showing him the pictures thereof, but it is far more difficult to teach him words of an abstract signification.

That the deaf mute must be more successful in forming abstract ideas upon the basis of spoken language, than can be effected through the method of signs, the language of which is devoid of prepositions, conjunctions, pronouns, tenses, moods, etc., is too obvious to require argument, not to mention all the other reasons why the system of teaching him to speak is preferable to that of signs and the manual alphabet.

It must be very gratifying to all the friends of deaf mutes that the American instructors now subject the method of articulation to the test of thorough, systematic experiment. In 1867, the "Institution for the Improved Instruction of Deaf Mutes," was established in New York city, and this system there introduced. The last annual report of this institution presents facts, that give evidence not only of its past prosperity, but are justly encouraging to its hopes for the future. Last winter the president and principal of the institution proceeded to Albany, taking with them a few of their pupils, in order to illustrate the method used in their school before the members of the Senate and Assembly in the State of New York. This they did with such success as to excite general surprise, consequently a bill was passed in both houses in the month of April, placing this institution upon the same legal basis with the old New York institution. Soon after, the State donated to that noble institution the sum of $10,000 to enable it to receive as many private, State, and county pupils as may be intrusted to their care.

For the sake of the large and unfortunate class of deaf mutes, we wish that this system may soon be adopted throughout all the United States.

